# Digital evaluation of the postoperative effects of orthognathic surgery on mandibular function in skeletal class III patients: a retrospective cohort study

**DOI:** 10.1186/s12903-026-10001-5

**Published:** 2026-10-15

**Authors:** Norbert Alexander Lang, Franziska Alina Lang, Claudia Mertes, Frank Hölzle, Peter Kessler, Michael Wolf, Rogerio Bastos Craveiro

**Affiliations:** 1 Department of Orthodontics, University Hospital RWTH Aachen, Pauwelsstraße 30, Aachen, 52074 Germany; 2 Department of Oral and Maxillofacial Surgery, University Hospital RWTH Aachen, Pauwelsstraße 30, Aachen, 52074 Germany; 3 Department of Cranio-Maxillofacial Surgery, Maastricht University Medical Center, Maastricht, 6229 HX Netherlands

**Keywords:** Interdisciplinary intervention, Combined orthodontic-orthognathic treatment, Digital axiographic recording system, 3D jaw motion analysis, Functional rehabilitation

## Abstract

**Objectives:**

Combined orthodontic-orthognathic intervention is the central approach for treating severe skeletal Class III malocclusion. This study quantifies longitudinally postoperative mandibular movement capacity as well as occlusal and condylar stability in patients with skeletal Class III using three-dimensional digital jaw-motion analysis.

**Materials and methods:**

The study included 20 patients with skeletal Class III malocclusion undergoing orthognathic surgery and 20 patients in the non-intervention control group. Functional measurements were obtained in the intervention group at a preoperative baseline after orthodontic decompensation (T0), and at the following five defined postoperative time points: T1 (2–3 weeks), T2 (6 weeks), T3 (3–4 months), T4 (6–9 months) and T5 (12–15 months). The patients followed a standardised surgical and rehabilitation protocol. The mandibular movement capacity and the occlusal and condylar stability during mouth opening, protrusion and laterotrusion were assessed using the Zebris JMA Optic three-dimensional jaw registration system. These were then compared with those of the control group.

**Results:**

Mandibular movement capacity decreased markedly during the early postoperative period and subsequently recovered in a movement-specific manner. At T5, mouth opening did not differ significantly from presurgical baseline (*p* = 0.224) or the control group, whereas condylar excursion during mouth opening remained significantly reduced compared with controls. Protrusion (*p* = 0.009) and right and left laterotrusion (both *p* = 0.002) exceeded baseline values and were comparable with controls at T5. Condylar recovery during protrusion and laterotrusion varied according to movement direction and side. Incisal-point stability improved from T0 to T5 across all movements (*p* = 0.002–0.031), although residual differences from controls remained after mouth opening (*p* = 0.003) and protrusion (*p* = 0.030). Condylar stability showed no significant longitudinal changes (all *p* ≥ 0.052), but no significant differences from controls remained at T5.

**Conclusion:**

Three-dimensional jaw movement analysis revealed movement-specific, time-dependent recovery over 12–15 months after orthognathic surgery in patients with skeletal Class III malocclusion. Selected protrusive and laterotrusive measures exceeded presurgical levels, whereas mouth opening recovery remained less complete. Overall, postoperative recovery is an ongoing adaptive process which can result in improved function compared to pre-surgery levels. This supports long-term functional monitoring and further comparative evaluation of treatment and retention protocols.

**Supplementary Information:**

The online version contains supplementary material available at https://doi.org/10.1186/s12903-026-10001-5.

## Introduction

Skeletal dysgnathias manifest themselves through significant deviations from normative skeletal and functional relationships and can significantly impair the facial profile and occlusion [1, 2]. Skeletal Class III malocclusion is considered one of the most complex orthodontic problems to diagnose and treat [3]. In addition to aesthetic limitations, skeletal dysgnathia potentially can lead to functional deficits, such as impaired chewing, breathing or speech, or relevant occlusal interferences. They are also associated with orofacial pain and a high prevalence of craniomandibular dysfunction [4]. According to the the index of orthognathic functional treatment need (IOFTN), patients with Class III malocclusion have a high clinical and functional need for orthognathic surgery [5]. Interdisciplinary treatment strategies consisting of orthognathic surgery and orthodontic therapy is a central approach in the treatment of pronounced skeletal dysgnathia and contribute significantly to improving quality of life [1, 6].

Careful postoperative follow-up is important to ensure long-term stability and patient adaptation. Rehabilitation strategies such as myofunctional therapy and guided physiotherapy help to retrain the masticatory muscles, tongue and lips [7, 8]. In addition, postsurgical orthodontics is essential for achieving successful and long-term stable outcomes [9–11]. This promotes proper function and reduces the risk of relapse. However, postoperative functional outcomes are still predominantly assessed using temporomandibular joint function, masticatory muscles and craniofacial morphology, and few have specifically investigated the functional adaptation of the mandible and occlusal and condylar stability caused by orthodontic-surgical treatments [12–15]. Furthermore, only a limited number of studies have employed homogeneous patient cohorts with a standardised treatment and follow-up protocols, and longitudinal data collection [8, 16, 17]. The lack of such standardisation therefore constitutes a relevant confounding factor when interpreting functional outcomes after orthognathic surgery.

The aim of this cohort study was to quantify the postoperative effects of orthognathic surgery on mandibular function over a follow-up period of up to 15 months. This was achieved by assessing mandibular movement capacity, condylar motion, and occlusal and condylar stability immediately before surgery and at five postoperative time points. These assessments were performed using three-dimensional digital jaw-motion analysis (Zebris JMA-Optic). The extent and temporal evolution of postoperative functional adaptation were characterised by analysing dynamic mandibular trajectories and end-position reproducibility, and these were then compared with those of a non-intervention control group. Our objective was to characterise the functional changes to the mandible after surgery, and to identify parameters that are relevant to postoperative rehabilitation.

## Materials and methods

This longitudinal, comparative retrospective cohort study analysed the incisal and condylar mandibular movements of an intervention group who received an interdisciplinary orthodontic-orthognathic treatment concept. All measurements were performed as part of a standardised clinical treatment protocol and were analysed retrospectively after completion of treatment. These movements were compared to those of a non-intervention control group using the three-dimensional digital jaw registration system Zebris JMA-Optic (Zebris Medical GmbH, Isny, Germany). The study was conducted in accordance with the World Medical Association’s Declaration of Helsinki on Medical Research. Ethical approval was obtained from the ethics committee of Medical Faculty of RWTH Aachen with the ethical registration number EK 25–302.

### Sample selection

**Fig. 1:**
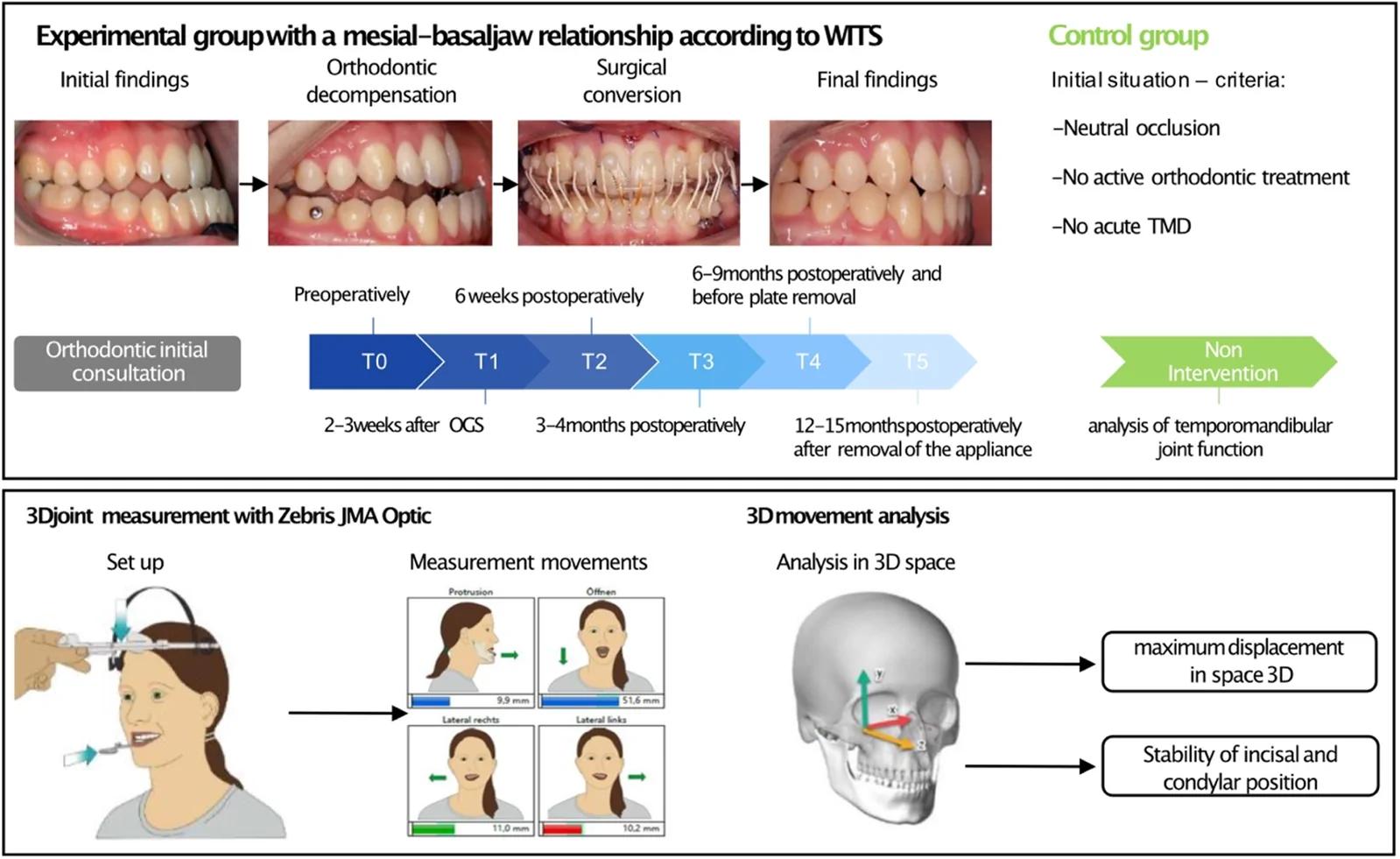
An overview of the study design, including a description of the experimental and control group, the three-dimensional jaw joint measurement and the analysis of lower jaw movements

The inclusion criteria for the intervention group undergoing an interdisciplinary orthodontic-orthognathic treatment concept at RWTH Aachen University Hospital between September 2023 and January 2026 were: (1) adult patients aged ≥ 18 years ; (2) a skeletal Class III malocclusion with a WITS appraisal < -1.0 mm; (3) combined orthodontic and orthognathic surgery treatment; (4) absence of craniofacial anomalies; (5) absence of facial trauma, mandibular condylar fractures, or maxillary bone defects. The inclusion criteria for the non- intervention control group comprised patients with (1) neutral occlusion (within a quarter of the premolar width), (2) no current orthodontic treatment or indication for treatment, (3) no temporomandibular joint dysfunction or movement limitations. No eligibility restrictions were applied based on sex. (Fig. 1).

### Data acquisition

Mandibular movements were systematically compared and evaluated by the same practitioner using the Zebris JMA-Optic system. The measurement settings were kept consistent: the Frankfurt horizontal plane served as the reference plane; the condylar position was determined using the C-positioning bow. To record mandibular movements, a T-attachment (paraocclusal tray) was first customised manually to the mandibular arch using orthopaedic forceps and then attached vestibular to the mandibular front teeth using bis-acrylic composite Luxatemp Star A3 (DMG, Hamburg, Germany). The measurement was performed using the CMDfact interactor module. This module records the three-dimensional trajectory of the incisal edge and the condylar kinematics, including translations and rotations. Data were transferred to WinJaw + 3.0 evaluation software (Zebris Medical GmbH, Isny, Germany), and all movement data were digitally archived and comprehensively analysed after each measurement. Three-dimensional mandibular movement was recorded in relation to a Cartesian coordinate system based on a reference plane defined parallel to the joint-orbital axis. The midpoint of the line connecting the centres of the condyles served as the origin of the three-dimensional spatial coordinate system. The X-axis served as the transverse direction connecting the centres of the condyles, the Y-axis served as the vertical superior-inferior direction, and the Z-axis served as the sagittal anterior-posterior direction.

The participants were given an introduction, after which the active measurements were taken as follows: (1) Mandibular movements should be performed separately for each individual movement, with tooth contact. (2) All movements should be performed to their fullest extent. (3) The movement should always begin and end in the habitual intercuspation. These instructions were given to record the functional movement paths and differentiate between occlusal influences on the movement pattern of the lower jaw. To obtain measurements for the functional analysis, all patients had to perform mandibular movements along four main movements: (1) mouth opening; (2) protrusion; and (3) right and (4) left laterotrusion. These movements were performed three times, after which the average measurements were calculated (Fig. 1). Participants were advised to sit upright with their head unsupported and to look straight ahead.

Functional measurements were recorded at the following times: Initial functional baseline measurement was obtained after the completion of orthodontic decompensation and before orthognathic surgery (T0); 2–3 weeks after orthognathic surgery (T1); 6 weeks postoperatively (T2); 3–4 months postoperatively (T3); 6–9 months postoperatively (T4) and 12–15 months postoperatively after removal of the multibracket appliance (T5). Additionally, as part of the standard preoperative planning and functional evaluation process, all patients underwent a clinical functional examination and routine panoramic radiography and lateral cephalometry. The control group was measured during a check-up appointment (TC), which serves as a functional reference. (Fig. 1)

Positive x, y and z coordinates corresponded to movements in the right lateral, cranial and anterior directions respectively, while negative values represented left lateral, caudal and posterior shifts. The maximum three-dimensional displacement in space (S; in mm) was calculated as the result of the translational movements along the X, Y, and Z axes (in mm). The following formula was used to determine the total vectorial movement [18, 19]:

$$\:S\:=\:\sqrt{\left(\left({X}^{2}\right)+\left({Y}^{2}\right)+\left({Z}^{2}\right)\right)}$$

### Outcome measures

The following functional parameters of each mandibular movement were analysed: (1) maximum displacement in space of the incisal point (IS) representing the movement capacity; (2) maximum displacement in space of the right (RCS) and left condyle (LCS) representing the translatory movement capacity of the condyles; (3) occlusal (incisal) stability, describing the deviation between the starting and ending points of the incisal point of the movements and thus the occlusal centring [20]; (4) condylar stability, defined as the stability of the condylar position at the beginning, and at the end of mandibular movement [20]. The principal endpoint was the longitudinal change in mandibular movement capacity, assessed by the maximum spatial displacement of the incisal point. Condylar movement capacity and occlusal and condylar stability were considered additional functional outcomes. The unit of measurement for the results is millimetres (mm).

A secondary analysis compared these functional parameters between the treated cohort and the non-intervention control group.

### Specific treatment and rehabilitation protocol

Preoperative orthodontic decompensation (i.e. intramaxillary treatment of the compensated condition) was performed at the Department of Orthodontics at RWTH Aachen University Hospital. Following consultation with the lead surgeons, the patient was cleared for surgery.

All orthognathic surgical procedures were conducted by two senior surgeons at the Department of Oral and Maxillofacial Surgery of the same university hospital, employing comparable surgical techniques. All patients were treated within a standardised interdisciplinary orthodontic–surgical protocol. Fixed multi-bracket appliances were used for pre-surgical orthodontic treatment. Surgical correction of skeletal Class III malocclusion was achieved either by isolated maxillary advancement (Le Fort I osteotomy) or by a bimaxillary procedure combining maxillary advancement with mandibular setback using bilateral sagittal split osteotomy. The surgical approach was selected based on individual dentofacial characteristics and treatment planning. The jaws were fixed using functionally stable osteosynthesis miniplates, which remained in place for six to nine months postoperatively. Postoperative rehabilitation followed a standardised seven-stage protocol coordinated between the Departments of Orthodontics and Oral and Maxillofacial Surgery (Table 1). During the initial phase (weeks 0–4), the focus was on stabilising the relationship between the maxilla and mandible, as well as the occlusion, after surgery. After the surgical splint has been removed, non-rigid stabilisation was achieved using full intermaxillary fixation (IMF) with extra heavy orthodontic elastics (diameter 1/8-inch, 6 oz). The initial phase involved monitoring for pain and infection, a dietary restriction, and the initiation of lymphatic drainage by qualified physiotherapists. The second phase (weeks 5–8) included continued full IMF with elastics and restriction of physical activity, as well as the initiation of structured physiotherapy. The third phase (month 3) involved reducing IMF with elastics progressively, transitioning to titanium–molybdenum alloy archwires, and initiating functional mobilisation. The fourth phase (month 4) involved progressively loading masticatory function while maintaining elastic guidance. During the fifth phase (month 5), IMF with elastics was limited to nocturnal use only. This was followed by further tapering of elastic support in the sixth phase (month 6). The final phase (months 6–9) involved removing the fixation plates and transitioning to orthodontic debonding and retention.

**Table 1:** Interdisciplinary treatment and rehabilitation protocol

Interdisciplinary treatment and rehabilitation protocol	
Time	Measures
Stage 1–1st month	0–2 weeks: • First appointment at the Department of Orthodontics after discharge and inspection of the surgical splint • Removal of the surgical splint 1–2 weeks postoperatively by the Department of Oral and Maxillofacial Surgery (OMS) • Weekly orthodontic check-up • Full IMF (intermaxillary fixation) using elastics • Liquid food diet for 3 weeks • Antibiotic therapy prescribed by OMS • Analgesic medication as required • Removal of absorbable sutures from postoperative day 10 by OMS 3–4 weeks: • Weekly orthodontic check-up • Full IMF using elastics • Soft/pureed food for 3 weeks • Initiation of lymphatic drainage
Stage 2- 2nd month	• Orthodontic check-up every 2 weeks, full IMF • Soft diet for 3 weeks • Avoidance of physical activity until this stage • Physiotherapy is recommended from week 6 onwards
Stage 3- 3rd month	• Orthodontic check-up every 4 weeks • Full IMF at night and reduced lateral elastics during the day • Transition to titanium-molybdenum alloy (TMA) archwires
Stage 4–4th month	• Single lateral elastics at night and during the day • Gradual return to full masticatory function • Contact sports and high-impact physical activity remain restricted
Stage 5–5th month	• Lateral elastics at night only
Stage 6–6th month	• Lateral elastics every second to third night
Stage 7–6-9 months	• Removal of osteosynthesis plates by OMS • Optional aesthetic corrections (e.g. nose or chin), if indicated • Debonding and initiation of retention possible from this stage

### Reliability

Repeated measurements were obtained from 20 non-intervention participants, with an interval of two to four weeks between sessions. Intra-rater reliability was evaluated using intraclass correlation coefficients (ICCs) with 95% confidence intervals for all measurement parameters between the appointments. ICC estimates were computed based on a single measurement, absolute-agreement, two-way mixed-effects model, and were calculated using IBM SPSS Statistics 29 software (IBM, Armonk, NY, USA) for the various movements. The following cut-off points were used to interpret the ICC values: <0.5: poor reliability; 0.5–0.75: moderate reliability; 0.75–0.9: good reliability; and > 0.9: excellent reliability [21].

### Power analysis

The sample size was calculated a priori using G*Power version 3.1.9.7, based on a two-tailed t-test for the difference between two independent means (α = 0.05; power [1 − β] = 0.80; allocation ratio 1:1). The expected effect size (d = 1.37) was derived from the means and standard deviations reported for the corresponding parameter in a methodologically comparable study [22], resulting in a required sample size of ten participants per group (achieved power, 0.83).

### Statistical analysis

Statistical analyses were performed in R 4.6.1 (R Foundation for Statistical Computing, Vienna, Austria), and figures were prepared using R 4.6.1 and Prism version 10.5.0 (GraphPad Software). Longitudinal changes in functional parameters were analysed using linear mixed-effects models (LMM) fitted by restricted maximum likelihood, with measurement time point (T0–T5) included as a categorical fixed effect and a random intercept for each patient, corresponding to a compound symmetry covariance structure. F-tests of the fixed effect of time were performed using the Satterthwaite approximation for the degrees of freedom. As a sensitivity analysis, the compound symmetry structure was compared with a first-order autoregressive [AR(1)] covariance structure using the Akaike and Bayesian information criteria under maximum likelihood estimation; the resulting conclusions were unchanged. Model residuals were assessed using Shapiro–Wilk tests and Q–Q plots. For parameters showing non-normally distributed residuals, the overall time effect was additionally assessed using Friedman tests in complete cases. Post-hoc comparisons of each postoperative time point with T0 were performed based on estimated marginal means, with Holm adjustment applied to the resulting p-values. The mixed-effects models incorporated all available observations and accommodated incomplete repeated-measures data under a missing-at-random (MAR) assumption. Secondary cross-sectional comparisons between the intervention cohort and the non-intervention control group at T0 and T5 were performed using Mann–Whitney U (MWU) tests, with Holm adjustment applied across the parameters within each parameter family at each time point. All tests were two-sided, and *p* < 0.05 was considered statistically significant. Two sensitivity analyses were performed. First, all longitudinal mixed-effects models were refitted with sex included as an additional fixed covariate, in order to assess the robustness of the time effect. Second, potential selection bias relating to missing T1 measurements was evaluated by comparing T0 range-of-motion parameters between patients with and without T1 data, using Mann–Whitney U tests with Holm correction.

## Results

**Fig. 2:**
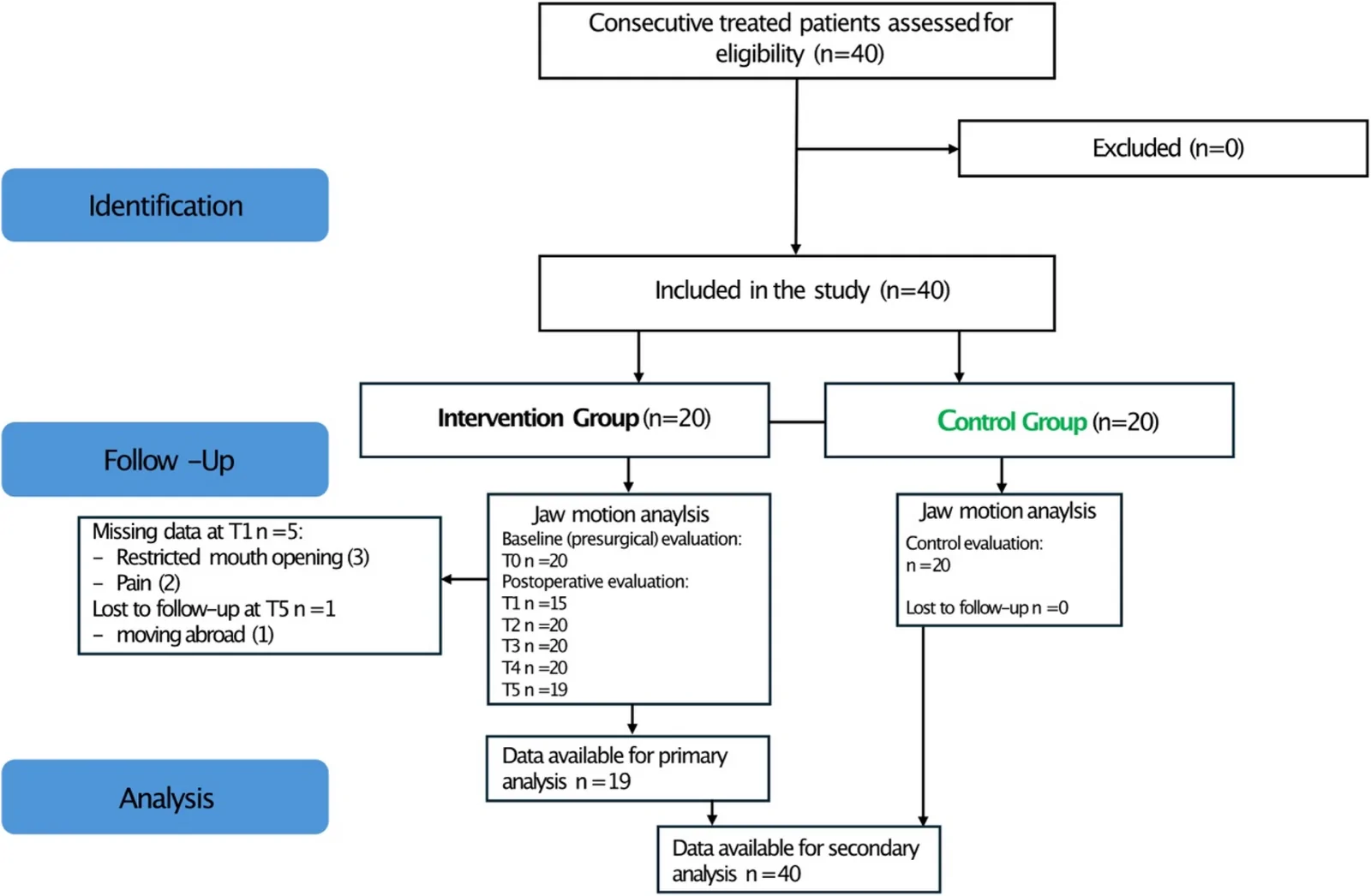
Study flowchart of patient cohort

A total of 20 consecutively treated adult patients (seven females and 13 males, with a mean age of 26.53 years, ± 9.18 years) with skeletal Class III malocclusion according to the WITS appraisal (mean − 7.52 mm ± 3.8) were included in the analysis. Of these, 15 patients (75%) underwent bimaxillary surgery and five (25%) underwent isolated maxillary advancement. Participant flow is reported in the cohort study flow chart. (Fig. 2).

The non-intervention group (ten females and ten males, with a mean age of 28.30 years, ± 3.23 years) underwent a standardised, three-dimensional mandibular movement analysis at two measurement points. The control cohort (*n* = 20) showed a mean overjet of 2.00 ± 0.45 mm and a mean overbite of 1.98 ± 0.75 mm; all participants had Angle Class I occlusion, a dental midline deviation of < 1 mm, and no signs or symptoms of TMD. The treated and non-intervention cohorts did not differ significantly in age or sex distribution (*p* = 0.424 and *p* = 0.523, respectively).

For the operated group, functional assessment of mandibular movement was performed at a total of six clearly defined points (± 1 week) in time during the course of therapy: after completion of orthodontic decompensation (T0; *n* = 20), 2–3 weeks postoperatively after removal of intermaxillary surgical splint (T1; *n* = 15), after six weeks (T2; *n* = 20), after three months (T3; *n* = 20), after six to nine months (T4; *n* = 20), immediately before the planned plate removal, and after completion of orthodontic fine adjustment and appliance removal in the period 12–15 months postoperatively (T5; *n* = 19). The entire study cohort of 20 patients was included in the analysis at T0, T2, T3 and T4. As shown in Fig. 2, the apparent reduction in sample size at certain time points (e.g. 15 patients at T1, with five patients missing due to pain and restricted mouth opening; and 19 patients at T5, with one patient missing at this time point) is due to missing measurements, rather than the exclusion of participants. (Fig. 2).

### ICC analysis

Intra-rater reliability showed consistently excellent reproducibility of the measurements, underscoring the robustness of the data collected. For mandibular movement, the inter-appointment ICCs indicated excellent reliability for all primary measurement parameters (> 0.9).

### Functional gains in mandibular movements

**Fig. 3:**
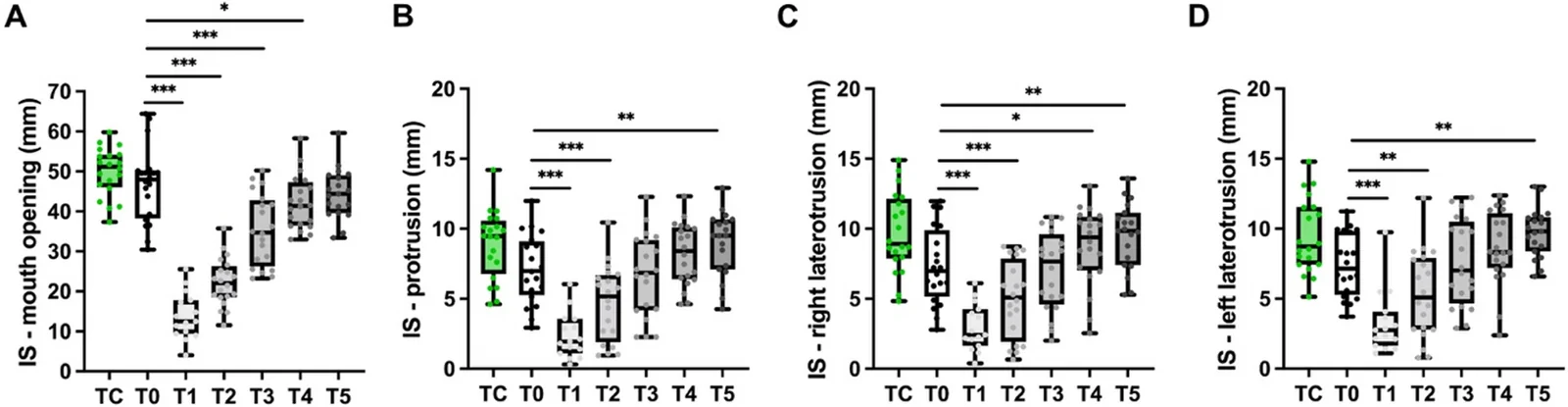
Movement capacity of mouth opening, protrusion and laterotrusion. Maximum spatial displacement of the Incisal point (IS) for mouth opening (**A**), protrusion (**B**), right laterotrusion (**C**), and left laterotrusion (**D**). Measurements are shown for the non-intervention control group (TC) and the surgically treated cohort at T0 (after orthodontic decompensation), T1 (2–3 weeks postoperatively), T2 (6 weeks), T3 (3–4 months), T4 (6–9 months), and T5 (12–15 months). Asterisks indicate post hoc comparisons of estimated marginal means between T0 and T1–T5 derived from the linear mixed-effects models, with Holm adjustment for multiple testing within each outcome (* p < 0.05, ** p < 0.01, and *** p < 0.001). Comparisons between the non-intervention control group and the treated cohort at T0 and T5 are reported separately in Table 2

Preoperatively, incisal-point movement capacity did not differ significantly between patients with skeletal Class III malocclusion and the control group for any movement ($\:{p}_{\mathrm{M}\mathrm{W}\mathrm{U}}\ge\:0.095$; Fig. 3A–D; Table 2). All movements decreased markedly immediately after surgery at T1 compared with T0 (all $\:{p}_{\mathrm{L}\mathrm{M}\mathrm{M},\mathrm{H}\mathrm{o}\mathrm{l}\mathrm{m}}<0.001$), followed by progressive functional recovery. Maximum mouth opening remained significantly reduced through T4 ($\:{p}_{\mathrm{L}\mathrm{M}\mathrm{M},\mathrm{H}\mathrm{o}\mathrm{l}\mathrm{m}}=0.031$) but no longer differed from baseline at T5 ($\:{p}_{\mathrm{L}\mathrm{M}\mathrm{M},\mathrm{H}\mathrm{o}\mathrm{l}\mathrm{m}}=0.224$) or from the control group ($\:{p}_{\mathrm{M}\mathrm{W}\mathrm{U}}=0.067$; Fig. 3A). Protrusive and laterotrusive movements had returned to baseline levels by T3 (all $\:{p}_{\mathrm{L}\mathrm{M}\mathrm{M},\mathrm{H}\mathrm{o}\mathrm{l}\mathrm{m}}\ge\:0.456$) and significantly exceeded their preoperative levels at T5 (protrusion: $\:{p}_{\mathrm{L}\mathrm{M}\mathrm{M},\mathrm{H}\mathrm{o}\mathrm{l}\mathrm{m}}=0.009$; right laterotrusion: $\:{p}_{\mathrm{L}\mathrm{M}\mathrm{M},\mathrm{H}\mathrm{o}\mathrm{l}\mathrm{m}}=0.002$; left laterotrusion: $\:{p}_{\mathrm{L}\mathrm{M}\mathrm{M},\mathrm{H}\mathrm{o}\mathrm{l}\mathrm{m}}=0.002$). At T5, none of these movements differed significantly from the control group (all $\:{p}_{\mathrm{M}\mathrm{W}\mathrm{U}}=1.000$; Fig. 3B–D; Table 2). Thus, protrusive and laterotrusive mandibular movements demonstrated significant functional gains relative to the preoperative baseline (Supplementary Tables 1 and 3).

**Table 2:** A comparison of movement capacity and functional occlusal stability between the control and experimental groups at T0 and T5

Parameters	Site	Control to T0 Holm-adjusted *p*-value	Control to T5 Holm-adjusted *p*-value
movement capacity			
	incisal point	0.379	0.067
mouth opening	right condyle	0.095	0.044 *
	left condyle	0.154	0.003 **
	incisal point	0.147	1.000
protrusion	right condyle	0.037 *	1.000
	left condyle	0.044 *	1.000
	incisal point	0.095	1.000
right laterotrusion	right condyle	1.000	1.000
	left condyle	0.044 *	1.000
	incisal point	0.134	1.000
left laterotrusion	right condyle	0.095	1.000
	left condyle	1.000	1.000
Functional occlusal stability			
	incisal point	< 0.001 ***	0.003 **
mouth opening	right condyle	0.003 **	0.253
	left condyle	0.036 *	0.664
	incisal point	< 0.001 ***	0.030 *
protrusion	right condyle	0.054	0.101
	left condyle	0.006 **	0.184
	incisal point	< 0.001 ***	0.956
right laterotrusion	right condyle	0.059	0.982
	left condyle	0.059	0.982
	incisal point	0.001 **	0.664
left laterotrusion	right condyle	0.026 *	0.567
	left condyle	0.028 *	0.380

Values are Holm-adjusted *p*-values from cross-sectional comparisons of the experimental group with the control group at T0 and T5. Holm adjustment was performed separately for each parameter family and time point (12 outcomes each). Significance levels are shown directly after each *p*-value: * *p* < 0.05, ** *p* < 0.01, and *** *p* < 0.001.; T0, baseline; T5, 12–15 months postoperatively after removal of the multibracket appliance follow-up

### Functional improvements in condylar movements

**Fig. 4:**
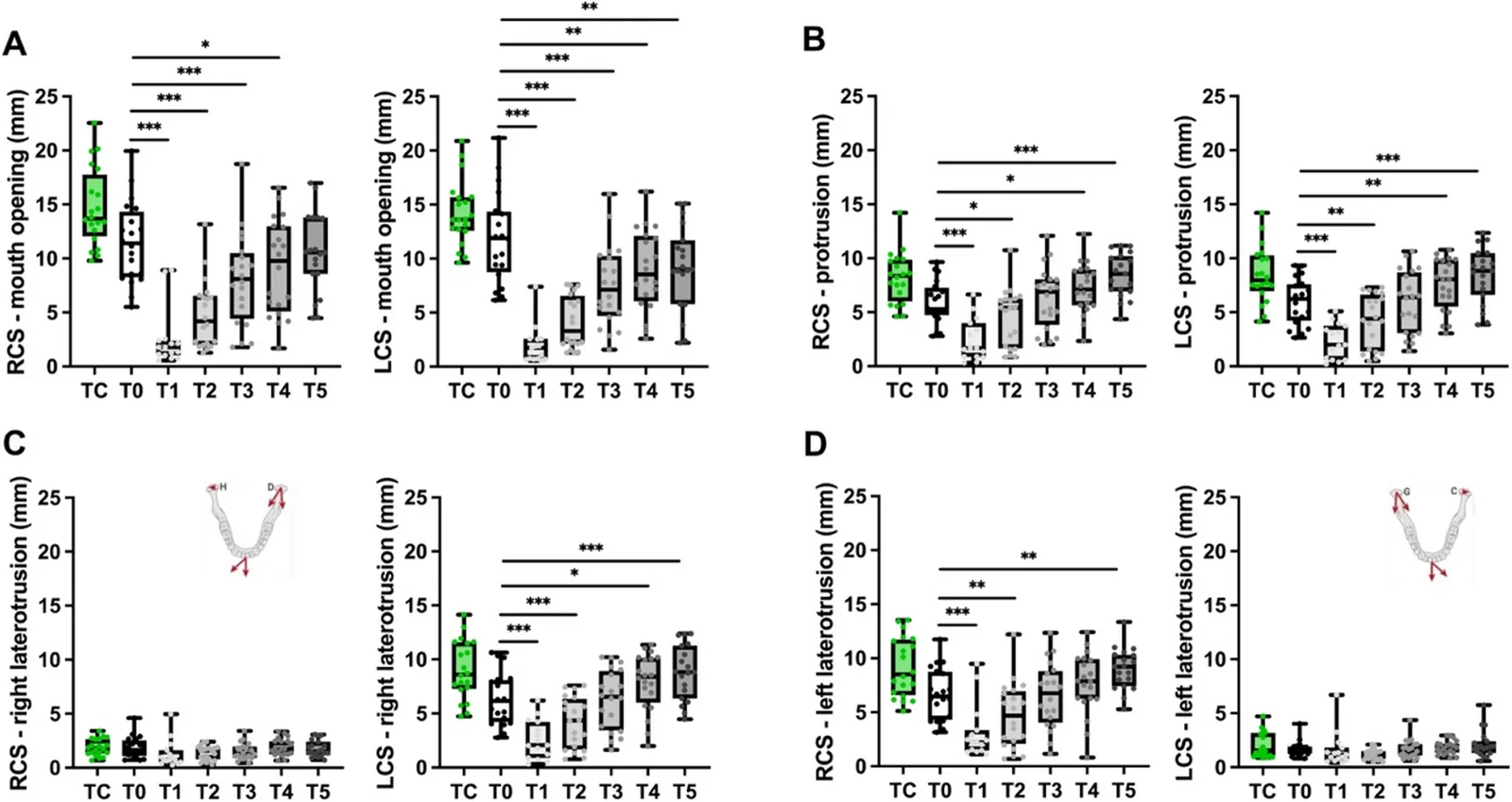
Movement capacity of the condyles during mouth opening, protrusion and laterotrusion. Maximum spatial displacement of the right (RCS) and left (LCS) condyle for mouth opening (**A**), protrusion (**B**), right laterotrusion (**C**), and left laterotrusion (**D**). Measurements are shown for the non-intervention control group (TC) and the surgically treated cohort at T0 (after orthodontic decompensation), T1 (2–3 weeks postoperatively), T2 (6 weeks), T3 (3–4 months), T4 (6–9 months), and T5 (12–15 months). Asterisks indicate post hoc comparisons of estimated marginal means between T0 and T1–T5 derived from the linear mixed-effects models, with Holm adjustment for multiple testing within each outcome (* p < 0.05, ** p < 0.01, and *** p < 0.001). Comparisons between the non-intervention control group and the treated cohort at T0 and T5 are reported separately in Table 2

Preoperatively, protrusive movement capacity was significantly lower than in the control group at both condyles (RCS: $\:{\boldsymbol{p}}_{\mathbf{M}\mathbf{W}\mathbf{U}}=0.037$; LCS: $\:{\boldsymbol{p}}_{\mathbf{M}\mathbf{W}\mathbf{U}}=0.044$). A significant deficit was also observed at the left condyle during right laterotrusion ($\:{\boldsymbol{p}}_{\mathbf{M}\mathbf{W}\mathbf{U}}=0.044$), whereas the remaining condylar comparisons were not significant (Fig. 4B–D; Table 2). The high-amplitude condylar excursions decreased markedly at T1 compared with T0 (all $\:{\boldsymbol{p}}_{\mathbf{L}\mathbf{M}\mathbf{M},\mathbf{H}\mathbf{o}\mathbf{l}\mathbf{m}}<0.001$), followed by progressive recovery. During mouth opening, the right condylar excursion no longer differed from baseline at T5 ($\:{\boldsymbol{p}}_{\mathbf{L}\mathbf{M}\mathbf{M},\mathbf{H}\mathbf{o}\mathbf{l}\mathbf{m}}=0.225$), whereas the left condylar excursion remained reduced ($\:{\boldsymbol{p}}_{\mathbf{L}\mathbf{M}\mathbf{M},\mathbf{H}\mathbf{o}\mathbf{l}\mathbf{m}}=0.002$). Both condyles continued to show reduced mouth-opening capacity compared with the control group at T5 (RCS: $\:{\boldsymbol{p}}_{\mathbf{M}\mathbf{W}\mathbf{U}}=0.044$; LCS: $\:{\boldsymbol{p}}_{\mathbf{M}\mathbf{W}\mathbf{U}}=0.003$; Fig. 4A; Table 2). By contrast, protrusive movement at T5 significantly exceeded the preoperative level at both condyles (RCS and LCS: $\:{\boldsymbol{p}}_{\mathbf{L}\mathbf{M}\mathbf{M},\mathbf{H}\mathbf{o}\mathbf{l}\mathbf{m}}<0.001$). The high-amplitude condylar excursions during right and left laterotrusion also significantly exceeded baseline (LCS during right laterotrusion: $\:{\boldsymbol{p}}_{\mathbf{L}\mathbf{M}\mathbf{M},\mathbf{H}\mathbf{o}\mathbf{l}\mathbf{m}}<0.001$; RCS during left laterotrusion: $\:{\boldsymbol{p}}_{\mathbf{L}\mathbf{M}\mathbf{M},\mathbf{H}\mathbf{o}\mathbf{l}\mathbf{m}}=0.001$), whereas the corresponding low-amplitude condylar paths remained unchanged. At T5, none of the protrusive or laterotrusive condylar movements differed significantly from the control group (all $\:{\boldsymbol{p}}_{\mathbf{M}\mathbf{W}\mathbf{U}}=1.000$; Fig. 4B–D; Table 2). Overall, condylar movement capacity during protrusion and laterotrusion showed significant functional improvement relative to baseline (Supplementary Tables 1 and 3).

### Functional occlusal stability

**Fig. 5:**
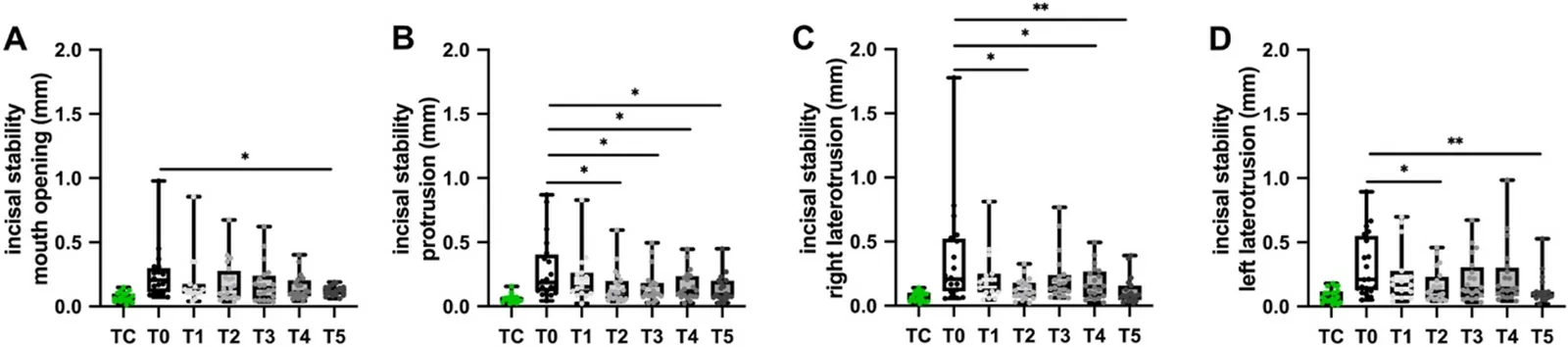
Functional occlusal stability after mouth opening, protrusion and laterotrusion. Incisal point stability, representing functional occlusal stability, for mouth opening (**A**), protrusion (**B**), right laterotrusion (**C**), and left laterotrusion (**D**). Measurements are shown for the non-intervention control group (TC) and the surgically treated cohort at T0 (after orthodontic decompensation), T1 (2–3 weeks postoperatively), T2 (6 weeks), T3 (3–4 months), T4 (6–9 months), and T5 (12–15 months). Asterisks indicate post hoc comparisons of estimated marginal means between T0 and T1–T5 derived from the linear mixed-effects models, with Holm adjustment for multiple testing within each outcome (* p < 0.05, ** p < 0.01, and *** p < 0.001). Comparisons between the non-intervention control group and the treated cohort at T0 and T5 are reported separately in Table 2

At T0, incisal-point stability was significantly lower in the operated group than in the control group for all movements (mouth opening, protrusion, and right laterotrusion: $\:{p}_{\mathrm{M}\mathrm{W}\mathrm{U}}<0.001$; left laterotrusion: $\:{p}_{\mathrm{M}\mathrm{W}\mathrm{U}}=0.001$; Fig. 5; Table 2). Despite some fluctuations during postoperative rehabilitation, incisal-point stability improved significantly from T0 to T5 for mouth opening ($\:{p}_{\mathrm{L}\mathrm{M}\mathrm{M},\mathrm{H}\mathrm{o}\mathrm{l}\mathrm{m}}=0.031$), protrusion ($\:{p}_{\mathrm{L}\mathrm{M}\mathrm{M},\mathrm{H}\mathrm{o}\mathrm{l}\mathrm{m}}=0.016$), right laterotrusion ($\:{p}_{\mathrm{L}\mathrm{M}\mathrm{M},\mathrm{H}\mathrm{o}\mathrm{l}\mathrm{m}}=0.002$), and left laterotrusion ($\:{p}_{\mathrm{L}\mathrm{M}\mathrm{M},\mathrm{H}\mathrm{o}\mathrm{l}\mathrm{m}}=0.006$; Fig. 5A–D). At T5, incisal-point stability remained significantly lower than in the control group after mouth opening ($\:{p}_{\mathrm{M}\mathrm{W}\mathrm{U}}=0.003$) and protrusion ($\:{p}_{\mathrm{M}\mathrm{W}\mathrm{U}}=0.030$), but was comparable between groups after right ($\:{p}_{\mathrm{M}\mathrm{W}\mathrm{U}}=0.956$) and left laterotrusion ($\:{p}_{\mathrm{M}\mathrm{W}\mathrm{U}}=0.664$; Table 2). These findings demonstrate an overall improvement in functional occlusal stability, with residual differences confined to mouth opening and protrusion (Supplementary Tables 2 and 3).

### Condylar stability

**Fig. 6:**
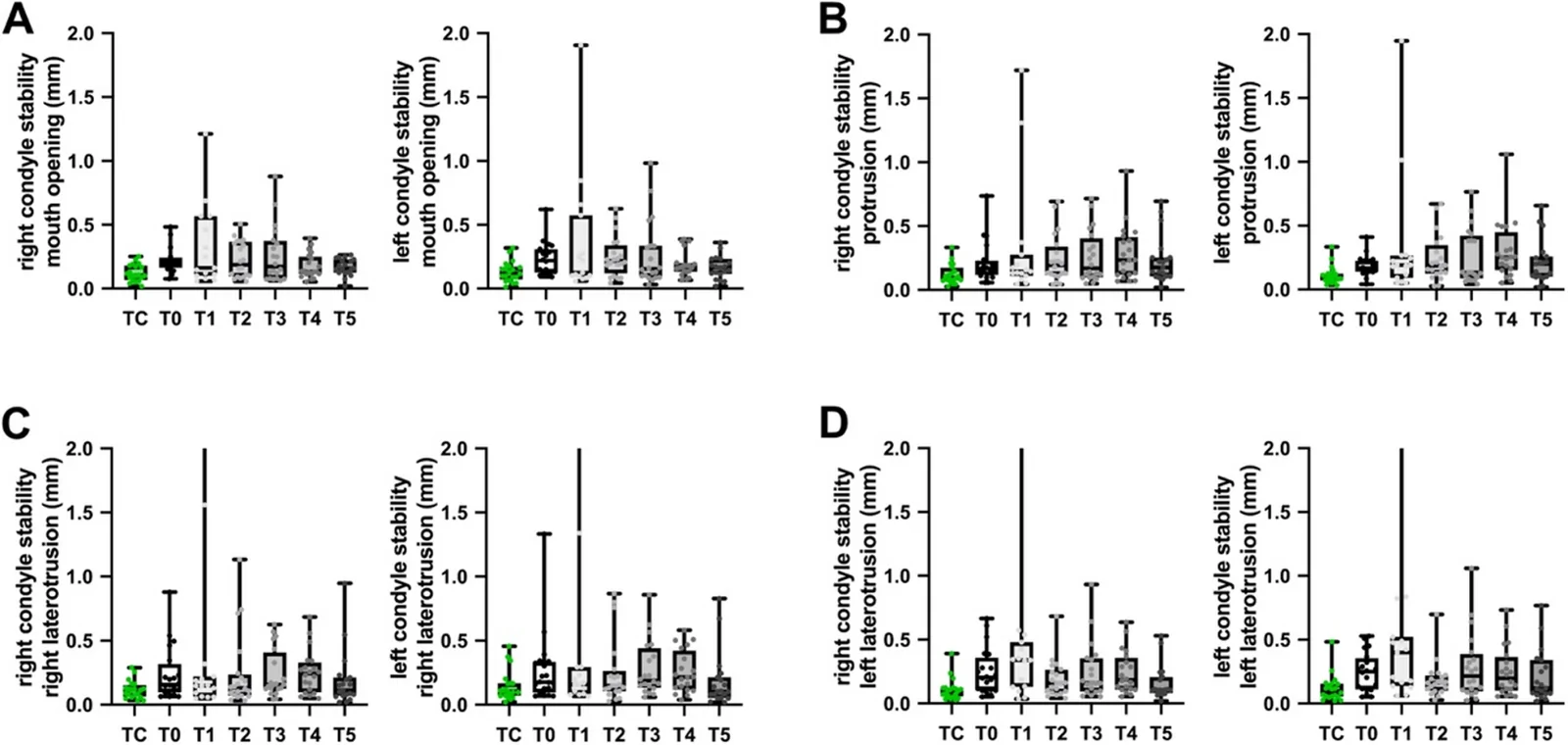
Condyle stability during mouth opening, protrusion and laterotrusion. Stability of the right (RCS) and left (LCS) condylar positions for mouth opening (**A**), protrusion (**B**), right laterotrusion (**C**), and left laterotrusion (**D**). Measurements are shown for the non-intervention control group (TC) and the surgically treated cohort at T0 (after orthodontic decompensation), T1 (2–3 weeks postoperatively), T2 (6 weeks), T3 (3–4 months), T4 (6–9 months), and T5 (12–15 months). Asterisks indicate post hoc comparisons of estimated marginal means between T0 and T1–T5 derived from the linear mixed-effects models, with Holm adjustment for multiple testing within each outcome (* p < 0.05, ** p < 0.01, and *** p < 0.001). Comparisons between the non-intervention control group and the treated cohort at T0 and T5 are reported separately in Table 2

At T0, condylar stability was significantly lower than in the control group at both condyles after mouth opening (RCS: $\:{\boldsymbol{p}}_{\mathbf{M}\mathbf{W}\mathbf{U}}=0.003$; LCS: $\:{\boldsymbol{p}}_{\mathbf{M}\mathbf{W}\mathbf{U}}=0.036$), at the left condyle after protrusion ($\:{\boldsymbol{p}}_{\mathbf{M}\mathbf{W}\mathbf{U}}=0.006$), and at both condyles after left laterotrusion (RCS: $\:{\boldsymbol{p}}_{\mathbf{M}\mathbf{W}\mathbf{U}}=0.026$; LCS: $\:{\boldsymbol{p}}_{\mathbf{M}\mathbf{W}\mathbf{U}}=0.028$; Fig. 6; Table 2). The remaining baseline comparisons were not significant. Although descriptive fluctuations were observed during postoperative rehabilitation, none of the condylar stability outcomes differed significantly from T0 at any postoperative time point (all $\:{\boldsymbol{p}}_{\mathbf{L}\mathbf{M}\mathbf{M},\mathbf{H}\mathbf{o}\mathbf{l}\mathbf{m}}\ge\:0.052$; Fig. 6A–D). At T5, no significant differences in condylar stability remained between the operated and control groups for any movement (all $\:{\boldsymbol{p}}_{\mathbf{M}\mathbf{W}\mathbf{U}}\ge\:0.101$; Table 2). Condylar stability therefore converged towards the control level without statistically significant longitudinal changes within the operated group (Supplementary Tables 2 and 3).

### Sensitivity analyses

Sex-adjusted sensitivity analyses revealed consistent longitudinal time effects across all 24 functional outcomes, suggesting that the primary findings remained robust even when adjusted for sex. After Holm correction, no significant T0 differences were observed for missing T1 data; T1 analyses were based on 15 patients, with all five patients missing at T1 returning for follow-up from T2 onwards.

## Discussion

The precise recording of mandibular movement sequences is a central component of modern functional diagnostics and therapy evaluation and is becoming increasingly important both in clinical application and in scientific research [23–25]. Optical jaw registration systems such as the Zebris JMA-Optic provide high-resolution, reproducible data for the quantitative analysis of complex jaw movements [26, 27]. Our results demonstrate the reliability of the measurements and confirm the applicability of the method in combined orthodontic-orthognathic treatments.

Carried out on a homogeneous cohort of Class III patients according to the WITS appraisal, using standardised surgical procedures, specific orthodontic treatments, and a rehabilitation protocol, this study provides detailed insights into the postoperative functional rehabilitation process. To evaluate as homogeneous a cohort as possible, the WITS appraisal was deliberately used instead of the ANB angle. In cephalometric analysis, the ANB angle, which measures the anteroposterior relationships between the maxilla and mandible in relation to the anterior cranial base, is subject to several significant limitations. A skeletal Class III was defined using the WITS appraisal, as this parameter allows a sagittal assessment of jaw misalignment that is less influenced by the morphology of the cranial base than angle measurements such as ANB, especially in patients with vertical discrepancies [28, 29, 30, 31].

Structural differences between patients with skeletal Class III malocclusion and individuals with dental Class I occlusion should be considered. Furthermore, as the control group was defined on the basis of dental and functional criteria, the presence of underlying skeletal discrepancies that have been dentally compensated cannot be completely ruled out. The control group therefore serves as a functional reference.

The preoperative baseline was determined following orthodontic decompensation. The baseline measurement was reflected by a reduced range of motion in protrusion and laterotrusion compared to the control group. However, the restriction in mouth opening was comparatively less pronounced. Following surgery, this deficit improved progressively over time. In several patients, functional ranges of motion exceeded preoperative baseline values and approached those of the control group within six to nine months postoperatively [32, 33].

Interestingly, recovery of movement at the incisal point was more pronounced than recovery of condylar excursion, particularly when opening the mouth. At the long-term follow-up, the maximum incisal opening approached the values of the non-intervention cohort, although a mean difference of approximately 5mm remained, while the condylar displacement remained comparatively reduced. This difference may suggest postoperative kinematic reorganization rather than persistent functional impairment, with mandibular rotation playing a greater role in compensating for reduced condylar translation. This is also evident in the literature, which shows that an increased mouth opening does not necessarily correspond to proportional condylar displacement [34–36]. Previous kinematic studies have demonstrated that the mouth opening capacity is predominantly determined by mandibular rotation and only weakly correlates with condylar translation. Therefore, restoring clinically relevant mandibular mobility does not necessarily require the underlying condylar movement pattern to be completely normalised.

By contrast, protrusive and laterotrusive movements exhibited similar recovery patterns at the incisal and condylar levels, achieving values comparable to those of the non-intervention control group by T5. This closer correspondence may be partly due to their stronger translational component: bilateral condylar translation during protrusion and predominant translation of the non-working condyle during laterotrusive movement.

Although no validated minimal clinically important difference has been established for mandibular movement after orthognathic surgery, evidence from populations with symptomatic temporomandibular disorder suggests that changes of several millimetres in mouth opening may exceed measurement variability and correspond to a perceived improvement by patients. However, these thresholds cannot be directly transferred to orthognathic patients. Furthermore, objective mandibular mobility does not necessarily correlate with patient-reported functional limitation [37, 38]. Nevertheless, recent evidence in skeletal Class III patients indicates that an earlier recovery of mandibular range of motion is associated with an earlier resumption of mouth-opening activities of daily living, which supports the functional importance of postoperative movement recovery [39].

The surgical procedure induces complex neuromuscular adaptations, which may initially evoke protective muscle guarding and transient hypomobility. Consistent with this response, early postoperative measurements showed marked reductions in all incisal-point movements and most condylar excursions [34, 40]. Protrusive and laterotrusive movements surpassed preoperative baseline values over time. The continuous functional increase observed up to the retention phase illustrates an ongoing adaptive rehabilitation process. Their approximation to the non-intervention control pattern therefore suggests a functional gain beyond simple restoration of the presurgical condition. By contrast, mouth opening showed less complete recovery at both the incisal and condylar levels. In patients undergoing mandibular setback, shortening of the effective sagittal mandibular length may have contributed to this residual limitation, alongside muscular and neuromuscular adaptation [40].

All patients in this study underwent the same standardised, strictly time-defined interdisciplinary surgical and postoperative rehabilitation protocol. Orthodontic measures aimed at promoting occlusal stabilisation, structured physiotherapy [41], and postoperative behavioural guidance (such as dietary restrictions) may have positively influenced both the temporal course of recovery, and the overall functional outcome observed in this study. In contrast, several previous studies report postoperative functional outcomes without providing detailed information on rehabilitation strategies, mobilisation timing or aftercare measures [8, 16, 17]. Heterogeneity in surgical techniques and follow-up protocols complicates comparisons of functional recovery across studies. In the present cohort, the standardised assessment protocol and predefined follow-up intervals nevertheless enabled a consistent characterisation of the time course of postoperative functional adaptation.

Relapse following orthognathic surgery represents one of the most clinically relevant and challenging complications [42, 43]. Long-term orthodontic stability is closely linked to the establishment of a stable and functionally integrated occlusion. As early as 1991, Pancherz emphasised the importance of stable intercuspation in reducing the risk of relapse [44, 45]. Further experimental evidence in rats suggests that adequate cusp–fossa contacts without occlusal interference may reduce post-orthodontic tooth displacement, as well as periodontal inflammatory and osteoclastic activity. However, its clinical transferability remains uncertain [46]. Importantly, the stability outcome assessed in the present study reflects the dynamic reproducibility of returning to habitual intercuspation and should not be interpreted as a direct measure or predictor of skeletal or dental relapse.

Overall, the findings indicate a movement-specific recovery of functional stability. Incisal-point stability improved across all movements, with no T5 differences from controls after laterotrusion but residual differences after mouth opening and protrusion, potentially reflecting movement-specific occlusal and neuromuscular adaptation. Condylar stability did not change significantly over time, although no between-group differences remained at T5. Jaschouz and Mehl reported a reproducibility of habitual intercuspation of 42± 34 μm using static registrations [47]. However, direct comparison is limited by the dynamic protocol and postoperative population of the present study. Given the lack of consensus regarding ideal functional occlusion and the methodological differences between static and dynamic assessments, the clinical significance of the deviations observed in the present study remains uncertain [48, 49].

Neuromuscular adaptation to altered tooth positions and occlusal contacts may continue beyond active orthodontic treatment [46, 50]. Orthognathic surgery can be expected to necessitate a prolonged neuromuscular adaptation phase, since altered functional demands, muscle stretching, and modified activation patterns require time-dependent structural and functional reorganisation of the jaw musculature [51]. During this phase, establishing stable intercuspation can reduce destabilising muscular forces and facilitate the development of a new functional equilibrium.

Therefore, the improvement in returning to habitual intercuspation that is specific to movement should be interpreted as progressive functional adaptation rather than as proof of complete occlusal normalisation. Although retention may provide a period during which neuromuscular and dentofacial soft-tissue adaptation can continue, its relationship with occlusal stability and long-term relapse requires further longitudinal investigation. The transient instability observed during the mid-postoperative period (e.g. around T3) may be influenced by external factors. Potential contributors include ongoing orthodontic treatment, a reduction in intermaxillary fixation duration, and the transition to titanium–molybdenum alloy archwires, all of which may affect early mechanical stabilisation, particularly when used in combination with elastics.

Condylar stability reflects the reproducibility with which the condyles assume the calibrated starting position and return to it following mandibular movement. Although deviations exceeding 0.3 mm have been interpreted as unstable, this recommendation-based cut-off has not been clinically validated for dynamic postoperative assessment [20, 52]. Incisal-point deviations tended to be smaller than condylar deviations from T1 onwards. This pattern was also observed in the control cohort. However, as these measures were not directly compared, it is possible that this reflects site-specific biomechanical or measurement characteristics. Therefore, while the findings are consistent with condylar positional reproducibility being comparable to that of the control group at the end of active treatment, they do not establish clinical temporomandibular joint stability. Nevertheless, dynamic stability assessment may contribute to evaluating functional adaptation following orthodontic and orthognathic interventions, although clinically meaningful thresholds require further validation.

These findings provide a basis for future validation studies and suggest that dynamic stability assessment may contribute to a better understanding of functional adaptation following orthodontic or surgical interventions.

## Limitations

The control group was defined based on dental Class I occlusion without cephalometric confirmation of skeletal relationships, which may limit structural comparability between groups. In accordance with the ALARA principle (‘As Low As Reasonably Achievable’), no X-rays were taken due to a lack of indication. Furthermore, the influence of factors such as the scope and quality of physiotherapy measures, individual differences in neuromuscular adaptation, and patient-related factors (e.g. motivation, fatigue, pain perception and sensory disturbances) was not systematically recorded. The baseline measurements were obtained preoperatively, rather than before orthodontic treatment began. Consequently, functional changes attributable to orthodontic preparation cannot be separated from the effects of surgery. However, as the primary aim of this study was to assess postoperative functional adaptation, the preoperative time point was considered the most relevant reference point for the longitudinal analysis. Different surgical approaches were not analysed separately. As these procedures may be associated with differing biomechanical and functional effects, this should be considered when interpreting the findings. This study did not record the removal of the plates and the associated postoperative reaction, or their influence on function. The single-centre design may limit generalisability; validation in larger multicentre cohorts is therefore warranted.

## Conclusions

Three-dimensional jaw movement analysis demonstrated movement-specific and time-dependent functional adaptation over 12–15 months following orthognathic surgery in patients with skeletal Class III malocclusion. Recovery continued beyond the early postoperative period, with protrusive and laterotrusive movements showing functional gains, whereas mouth opening remained less completely restored at both the incisal and condylar levels. Movement capacity and stability followed distinct trajectories, highlighting the complementary information provided by incisal and condylar measurements. Overall, the findings suggest that postoperative recovery represents an ongoing adaptive process and, for selected movements, may exceed simple restoration of the presurgical condition. Structured long-term functional monitoring appears warranted, while comparative studies are required to determine the specific contribution of postoperative treatment and retention protocols.

## Supplementary Information

Supplementary Material 1.

## Data Availability

The datasets used and/or analysed during the current study are available from the corresponding author upon reasonable request.
