# Supplementary material for: Digital evaluation of the postoperative effects of orthognathic surgery on mandibular function in skeletal class III patients: a retrospective cohort study
Source: BMC Oral Health. 2026 Oct 15;26:2203. doi: 10.1186/s12903-026-10001-5 (PMC13741542; doi:10.1186/s12903-026-10001-5)
Supplement: Supplementary file 1 [file 12903_2026_10001_MOESM1_ESM.docx]

**Digital evaluation of the postoperative effects of orthognathic surgery on mandibular function in skeletal Class III patients: a retrospective cohort study**

**Supplementary**

***Supplementary Table 1****.* Representation of the movement capacity for the four movement parameters: mouth opening; protrusion; laterotrusion right and laterotrusion left.

| **Movement** | | **Maximum movement capacity in millimetres** | | | | | | | | | | | | | |
| --- | --- | --- | --- | --- | --- | --- | --- | --- | --- | --- | --- | --- | --- | --- | --- |
|  |  | **Experimental group with a mesial-basal jaw relationship according to WITS** | | | | | | | | | | |  | **Control group** | |
|  |  | **T0** | | **T1** | | **T2** | | **T3** | | **T4** | | **T5** | | **TC** | |
|  | | Mean (SD) | CI 95% (lower to upper) | Mean (SD) | CI 95% (lower to upper) | Mean (SD) | CI 95% (lower to upper) | Mean (SD) | CI 95% (lower to upper) | Mean (SD) | CI 95% (lower to upper) | Mean (SD) | CI 95% (lower to upper) | Mean (SD) | CI 95% (lower to upper) |
| **MO*** | **IS*** | 46.61 (9.33) | 42.24 to 50.98 | 13.86 (5.813) | 10.64 to 17.08 | 22.28 (5.963) | 19.48 to 25.07 | 35.35 (8.930) | 31.17 to 39.52 | 42.23 (6.731) | 39.08 to 45.38 | 44.45 (6.221) | 41.45 to 47.44 | 49,92 (6.057) | 47.08 to 52.75 |
|  | **RCS*** | 11.47 (3.755) | 9.716 to 13.23 | 2.491 (2.656) | 1.020 to 3.962 | 4.821 (3.166) | 3.339 to 6.303 | 8.069 (4.245) | 6.082 to 10.06 | 9.196 (4.417) | 7.129 to 11.26 | 10.49 (3.691) | 8.709 to 12.27 | 14.69 (3.613) | 13.00 to 16.38 |
|  | **LCS*** | 11.86 (4.151) | 9.918 to 13.80 | 1.962 (1.777) | 0.9780 to 2.946 | 4.085 (2.428) | 2.948 to 5.221 | 7.463 (3.767) | 5.700 to 9.226 | 8.918 (3.729) | 7.173 to 10.66 | 9.198 (3.896) | 7.320 to 11.08 | 14.22 (3.008) | 12.82 to 15.63 |
| **P*** | **IS*** | 7.12 (2.450) | 5.970 to 8.263 | 2.371 (1.603) | 1.483 to 3.259 | 4.679 (2.694) | 3.418 to 5.939 | 6.690 (2.957) | 5.306 to 8.074 | 8.200 (2.226) | 7.160 to 9.242 | 8.941 (2.229) | 7.837 to 10.04 | 8.905 (2.466) | 7.751 to 10.06 |
|  | **RCS*** | 5.882 (1.966) | 4.962 to 6.802 | 2.395 (1.942) | 1.320 to 3.470 | 4.540 (2.677) | 3.286 to 5.793 | 6.302 (2.861) | 4.963 to 7.641 | 7.214 (2.338) | 6.120 to 8.308 | 8.415 (2.040) | 7.431 to 9.398 | 8.198 (2.355) | 7.096 to 9.301 |
|  | **LCS*** | 6.013 (2.095) | 5.032 to 6.993 | 2.321 (1.614) | 1.428 to 3.215 | 4.247 (2.463) | 3.094 to 5.400 | 5.925 (2.874) | 4.579 to 7.270 | 7.561 (2.422) | 6.428 to 8.695 | 8.420 (2.538) | 7.196 to 9.643 | 8.468 (2.593) | 7.254 to 9.681 |
| **L ri*** | **IS*** | 7.312 (2.733) | 6.033 to 8.591 | 2.962 (1.660) | 2.043 to 3.882 | 4.960 (2.755) | 3.670 to 6.249 | 7.011 (2.840) | 5.682 to 8.340 | 8.773 (2.777) | 7.473 to 10.07 | 9.509 (2.355) | 8.374 to 10.64 | 9.714 (2.844) | 8.383 to 11.05 |
|  | **RCS*** | 1.832 (0.9924) | 1.367 to 2.296 | 1.285 (1.255) | 0.5902 to 1.981 | 1.392 (0.6572) | 1.085 to 1.700 | 1.514 (0.7575) | 1.160 to 1.869 | 1.887 (0.7981) | 1.513 to 2.260 | 1.777 (0.7444) | 1.418 to 2.136 | 1.885 (0.772) | 1.524 to 2.446 |
|  | **LCS*** | 6.313 (2.366) | 5.205 to 7.420 | 2.566 (1.787) | 1.577 to 3.556 | 4.287 (2.360) | 3.149 to 5.425 | 6.295 (2.772) | 4.997 to 7.592 | 7.925 (2.658) | 6.681 to 9.169 | 8.708 (2.473) | 7.551 to 9.866 | 9.041 (2.720) | 7.768 to 10.30 |
| **L le*** | **IS*** | 7.419 (2.239) | 6.372 to 8.467 | 3.296 (2.248) | 2.051 to 4.541 | 5.331 (3.110) | 3.875 to 6.786 | 7.588 (3.091) | 6.141 to 9.035 | 8.629 (2.637) | 7.395 to 9.864 | 9.732 (1.750) | 8.888 to 10.58 | 9.476 (2.654) | 8.233 to 10.72 |
|  | **RCS*** | 6.681 (2.359) | 5.577 to 7.785 | 3.132 (2.524) | 1.734 to 4.529 | 4.679 (3.035) | 3.259 to 6.099 | 6.575 (3.022) | 5.161 to 7.990 | 7.685 (2.846) | 6.353 to 9.017 | 9.018 (1.906) | 8.100 to 9.937 | 9.188 (2.753) | 7.900 to 10.48 |
|  | **LCS*** | 1.700 (0.6827) | 1.380 to 2.019 | 1.615 (1.594) | 0.7323 to 2.497 | 1.193 (0.4747) | 0.9709 to 1.415 | 1.680 (0.8719) | 1.272 to 2.088 | 1.830 (0.6335) | 1.534 to 2.127 | 2.139 (1.248) | 1.537 to 2.740 | 1.721 (0.8407) | 1.338 to 2.104 |

MO: mouth opening; P: protrusion; L ri: laterotrusion right; L le: laterotrusion left; IS: displacement in space of the initial point; RCS: displacement in space of right condyle; LCS: displacement in space of left condyle

***Supplementary Table 2****.* Representation of the occlusal and condyle stability for the four movement parameters: mouth opening; protrusion; laterotrusion right and laterotrusion left.

| **Movement** | | **Deviation of ccclusal and condyle stability in millimetres** | | | | | | | | | | | | | |
| --- | --- | --- | --- | --- | --- | --- | --- | --- | --- | --- | --- | --- | --- | --- | --- |
|  |  | **Experimental group with a mesial-basal jaw relationship according to WITS** | | | | | | | | | | | | **Control group** | |
|  |  | **T0** | | **T1** | | **T2** | | **T3** | | **T4** | | **T5** | | **TC** | |
|  | | Mean (SD) | CI 95% (lower to upper) | Mean (SD) | CI 95% (lower to upper) | Mean (SD) | CI 95% (lower to upper) | Mean (SD) | CI 95% (lower to upper) | Mean (SD) | CI 95% (lower to upper) | Mean (SD) | CI 95% (lower to upper) | Mean (SD) | CI 95% (lower to upper) |
| **MO** | **IS** | 0.2488 (0.1999) | 0.1553 to 0.3424 | 0.1924 (0.2037) | 0.07962 to 0.3052 | 0.1925 (0.1600) | 0.1176 to 0.2674 | 0.1813 (0.1540) | 0.1092 to 0.2534 | 0.1495 (0.09611) | 0.1045 to 0.1944 | 0.1231 (0.0368) | 0,1053 to 0,1409 | 0.07127 (0.03587) | 0.05448 to 0.08806 |
|  | **RCS** | 0.2248 (0.09813) | 0.1788 to 0.2707 | 0.3257 (0.3226) | 0.1470 to 0.5044 | 0.2262 (0.1409) | 0.1602 to 0.2921 | 0.2600 (0.2220) | 0.1561 to 0.3639 | 0.1755 (0.09743) | 0.1299 to 0.2211 | 0.1691 (0.06575) | 0.1374 to 0.2008 | 0.1220 (0.06552) | 0.09134 to 0.1527 |
|  | **LCS** | 0.2328 (0.1305) | 0.1718 to 0.2939 | 0.3657 (0.4908) | 0.09396 to 0.6375 | 0.2489 (0.1587) | 0.1746 to 0.3232 | 0.2680 (0.2572) | 0.1477 to 0.3884 | 0.1904 (0.09946) | 0.1438 to 0.2369 | 0.1648 (0.08804) | 0.1224 to 0.2073 | 0.1329 (0.08009) | 0.09540 to 0.1704 |
| **P** | **IS** | 0.2869 (0.2404) | 0.1744 to 0.3994 | 0.2209 (0.1964) | 0.1122 to 0.3297 | 0.1517 (0.1365) | 0.0877 to 0.2155 | 0.1528 (0.1283) | 0.09279 to 0.2129 | 0.1592 (0.1155) | 0.1052 to 0.2132 | 0.1351 (0.1039) | 0.08505 to 0.1852 | 0.06061 (0.03040) | 0.04638 to 0.07484 |
|  | **RCS** | 0.2130 (0.1618) | 0.1373 to 0.2887 | 0.3358 (0.4919) | 0.06339 to 0.6082 | 0.2419 (0.1905) | 0.1527 to 0.3311 | 0.2588 (0.2035) | 0.1636 to 0.3541 | 0.2845 (0.2150) | 0.1839 to 0.3852 | 0.2349 (0.1870) | 0.1448 to 0.3251 | 0.1213 (0.07767) | 0.08499 to 0.1577 |
|  | **LCS** | 0.1877 (0.07887) | 0.1508 to 0.2246 | 0.3392 (0.5015) | 0.06144 to 0.6169 | 0.2415 (0.1843) | 0.1553 to 0.3278 | 0.2565 (0.2167) | 0.1551 to 0.3580 | 0.3005 (0.2306) | 0.1926 to 0.4084 | 0.2180 (0.1732) | 0.1345 to 0.3015 | 0.1132 (0.07145) | 0.07977 to 0.1467 |
| **L ri** | **IS** | 0.3587 (0.3993) | 0.1719 to 0.5456 | 0.2212 (0.2005) | 0.1102 to 0.3323 | 0.1261 (0.08097) | 0.08821 to 0.1640 | 0.2183 (0.1887) | 0.1300 to 0.3066 | 0.1772 (0.1282) | 0.1171 to 0.2372 | 0.1174 (0.1063) | 0.06617 to 0.1686 | 0.07141 (0.03489) | 0.05508 to 0.08774 |
|  | **RCS** | 0.2382 (0.2094) | 0.1402 to 0.3362 | 0.4180 (0.7751) | -0.01123 to 0.8472 | 0.2429 (0.2912) | 0.1066 to 0.3792 | 0.2387 (0.1887) | 0.1504 to 0.3270 | 0.2529 (0.1705) | 0.1730 to 0.3327 | 0.1909 (0.2220) | 0.08387 to 0.2979 | 0.1182 (0.06918) | 0.08582 to 0.1506 |
|  | **LCS** | 0.2677 (0.2832) | 0.1351 to 0.4002 | 0.4315 (0.7953) | -0.008907 to 0.8719 | 0.2510 (0.2557) | 0.1313 to 0.3707 | 0.2811 (0.2152) | 0.1804 to 0.3818 | 0.2567 (0.1652) | 0.1794 to 0.3341 | 0.1844 (0.2148) | 0.08085 to 0.2879 | 0.1458 (0.1166) | 0.09125 to 0.2004 |
| **L le** | **IS** | 0.3188 (0.2439) | 0.2047 to 0.4330 | 0.2452 (0.2191) | 0.1239 to 0.3665 | 0.1624 (0.1283) | 0.1023 to 0.2224 | 0.2110 (0.1768) | 0.1282 to 0.2938 | 0.2378 (0.2366) | 0.1271 to 0.3485 | 0.1307 (0.1162) | 0.07465 to 0.1867 | 0.08403 (0.05239) | 0.05951 to 0.1086 |
|  | **RCS** | 0.2495 (0.1797) | 0.1653 to 0.3336 | 0.4171 (0.5335) | 0.1216 to 0.7125 | 0.1900 (0.1469) | 0.1213 to 0.2587 | 0.2499 (0.2229) | 0.1456 to 0.3542 | 0.2433 (0.1671) | 0.1651 to 0.3215 | 0.1606 (0.1344) | 0.0958 to 0.2253 | 0.1105 (0.08421) | 0.07110 to 0.1499 |
|  | **LCS** | 0.2472 (0.1514) | 0.1763 to 0.3180 | 0.4715 (0.5280) | 0.1790 to 0.7639 | 0.1848 (0.1415) | 0.1185 to 0.2510 | 0.2883 (0.2632) | 0.1651 to 0.4115 | 0.2501 (0.1939) | 0.1594 to 0.3408 | 0.2142 (0.2033) | 0.1162 to 0.3122 | 0.1193 (0.1053) | 0.07000 to 0.1685 |

MO: mouth opening; P: protrusion; L ri: laterotrusion right; L le: laterotrusion left; IS: displacement in space of the initial point; RCS: displacement in space of right condyle; LCS: displacement in space of left condyle

***Supplementary Table 3****.* **Postoperative changes in mandibular movement capacity and stability compared with the preoperative baseline (T0)**

| **Parameters** | **Site** | **T1 - T0** | **T2 - T0** | **T3 - T0** | **T4 - T0** | **T5 - T0** |
| --- | --- | --- | --- | --- | --- | --- |
| **Movement capacity** | | | | | | |
| mouth opening | incisal point | **<0.001 ***** | **<0.001 ***** | **<0.001 ***** | **0.031 *** | 0,224 |
|  | right condyle | **<0.001 ***** | **<0.001 ***** | **<0.001 ***** | **0.014 *** | 0,225 |
|  | left condyle | **<0.001 ***** | **<0.001 ***** | **<0.001 ***** | **0.001 **** | **0.002 **** |
| protrusion | incisal point | **<0.001 ***** | **<0.001 ***** | 0,456 | 0,121 | **0.009 **** |
|  | right condyle | **<0.001 ***** | **0.040 *** | 0,433 | **0.040 *** | **<0.001 ***** |
|  | left condyle | **<0.001 ***** | **0.004 **** | 0,868 | **0.009 **** | **<0.001 ***** |
| right laterotrusion | incisal point | **<0.001 ***** | **<0.001 ***** | 0,612 | **0.031 *** | **0.002 **** |
|  | right condyle | 0,123 | 0,279 | 0,564 | 1,000 | 1,000 |
|  | left condyle | **<0.001 ***** | **<0.001 ***** | 0,975 | **0.011 *** | **<0.001 ***** |
| left laterotrusion | incisal point | **<0.001 ***** | **0.003 **** | 0,781 | 0,097 | **0.002 **** |
|  | right condyle | **<0.001 ***** | **0.003 **** | 0,856 | 0,179 | **0.001 **** |
|  | left condyle | 1,000 | 0,350 | 1,000 | 1,000 | 0,456 |
| **Stability** | | | | | | |
| mouth opening | incisal point | 0,414 | 0,414 | 0,394 | 0,110 | **0.031 *** |
|  | right condyle | 0,405 | 1,000 | 1,000 | 1,000 | 1,000 |
|  | left condyle | 0,418 | 1,000 | 1,000 | 1,000 | 1,000 |
| protrusion | incisal point | 0,204 | **0.028 *** | **0.028 *** | **0.028 *** | **0.016 *** |
|  | right condyle | 0,683 | 1,000 | 1,000 | 1,000 | 1,000 |
|  | left condyle | 0,309 | 1,000 | 1,000 | 0,468 | 1,000 |
| right laterotrusion | incisal point | 0,064 | **0.002 **** | 0,064 | **0.018 *** | **0.002 **** |
|  | right condyle | 0,702 | 1,000 | 1,000 | 1,000 | 1,000 |
|  | left condyle | 0,920 | 1,000 | 1,000 | 1,000 | 1,000 |
| left laterotrusion | incisal point | 0,289 | **0.022 *** | 0,160 | 0,289 | **0.006 **** |
|  | right condyle | 0,257 | 1,000 | 1,000 | 1,000 | 1,000 |
|  | left condyle | 0,052 | 1,000 | 1,000 | 1,000 | 1,000 |

Values are Holm-adjusted p-values from post hoc contrasts of estimated marginal means derived from linear mixed-effects models, comparing each postoperative time point (T1–T5) with baseline T0. The Holm correction was applied separately to the five contrasts within each outcome. Significance levels are indicated directly after each p-value: p < 0.05, p < 0.01, and p < 0.001. T0, baseline after orthodontic decompensation; T1, 2–3 weeks postoperatively; T2, 6 weeks postoperatively; T3, 3–4 months postoperatively; T4, 6–9 months postoperatively; T5, 12–15 months postoperatively following removal of the multibracket appliance.
